# Prevalence of Neurosyphilis in Patients with Acute Ischemic Stroke: A Cross-Sectional Screening Study in Thailand

**DOI:** 10.3390/tropicalmed11050117

**Published:** 2026-04-29

**Authors:** Chumpol Anamnart, Nawanwat Tepkidakarn

**Affiliations:** 1 Division of Neurology, Department of Medicine, Prapokklao Hospital, 38 Wat Mai, Mueang Chanthaburi District, Chanthaburi 22000, Thailand; 2 Division of Diagnostic Radiology, Department of Radiology, Prapokklao Hospital, 38 Wat Mai, Mueang Chanthaburi District, Chanthaburi 22000, Thailand; jeannynawanwat@gmail.com

**Keywords:** neurosyphilis, meningovascular syphilis, venereal disease research laboratory test, magnetic resonance vessel wall imaging

## Abstract

Meningovascular syphilis, a type of neurosyphilis, causes stroke and various types of myelopathy. In recent years, there has been an increase in the incidence of neurosyphilis. However, diagnosing neurosyphilis remains challenging due to the reliance on serum and cerebrospinal fluid (CSF) testing, which has low specificity and sensitivity. Magnetic resonance vessel wall imaging (MR-VWI), recently developed to identify vessel wall pathologies, may aid in diagnosing neurosyphilis. In this cross-sectional study, we performed systematic screening for syphilis in all 366 patients with acute ischemic stroke or transient ischemic attack admitted to our stroke unit. Further CSF analysis and MR-VWI were specifically conducted only on those with reactive serum venereal disease research laboratory (VDRL) or treponema pallidum particle hemagglutination assay (TPHA) tests to evaluate neurosyphilis. Serum screening was reactive in 5.7% (21/366) of patients; among these, the prevalence of likely neurosyphilis (defined by abnormal CSF pleocytosis or protein levels) was 2.2% (8/366). Within this group of eight patients, MR-VWI was technically feasible and thus performed in six cases. Although all CSF-VDRL tests were non-reactive, MR-VWI identified diagnostic evidence of meningovascular syphilis (concentric wall thickening and enhancement) in 33.3% (2/6) of symptomatic patients who underwent the scan. Neurosyphilis remains a critical, treatable cause of stroke that can affect older patients with established vascular risk factors. Our findings demonstrate that routine serum screening is essential, as traditional CSF-VDRL tests may yield false-negative results. MR-VWI serves as a valuable adjunct tool to provide objective evidence of active vasculitis, guiding the initiation of appropriate antibiotic therapy when laboratory results are inconclusive.

## 1. Introduction

Syphilis is an infection caused by the bacterium *Treponema pallidum* [1], and neurosyphilis is a complication of untreated syphilis that affects the central nervous system [1]. Recently, syphilitic infections, including neurosyphilis, have garnered renewed attention as their incidence has increased worldwide [2,3,4,5]. In Thailand, the resurgence of syphilis has emerged as a significant public health challenge. According to national surveillance data, the incidence rate of syphilis has surged nearly sixfold over the past decade, rising from 3.60 per 100,000 members of the population in 2013 to 20.93 per 100,000 members of the population in 2022. This dramatic epidemiological shift underscores the increasing risk of neurosyphilis-related complications, including meningovascular syphilis, within the general population [6]. The global resurgence of syphilis highlights an urgent clinical challenge where neurosyphilis often remains an under-recognized cause of ischemic stroke. Conventional diagnostic criteria heavily rely on CSF-VDRL, which frequently yields false-negative results, potentially leading to missed opportunities for curative antibiotic treatment. Therefore, evaluating the prevalence of this condition in a high-burden setting and investigating the role of advanced imaging like MR-VWI are not only clinically relevant but essential for refining diagnostic protocols and improving neurological outcomes in stroke patients.

The clinical manifestations of neurosyphilis can either be asymptomatic or symptomatic. If left untreated, the infection typically progresses from primary and secondary stages to tertiary syphilis, where serious complications such as cardiovascular and cerebrovascular diseases often manifest [7]. Meningovascular syphilis (MVS) is a specific form of neurosyphilis that can cause strokes and various types of myelopathy [8,9,10]. Typically, MVS occurs during the early stage of syphilitic infection; it can also be observed as a late form of neurosyphilis [11]. 

Neurosyphilis is diagnosed using serum and cerebrospinal fluid (CSF) tests, and elevated white blood cell (WBC) counts and protein levels in the CSF support the diagnosis [8]. Owing to its high specificity, the venereal disease research laboratory (VDRL) test is the gold standard for diagnosing neurosyphilis, with a reactive CSF-VDRL test confirming the diagnosis; however, its sensitivity is low (30–70%), and it varies with the stage of syphilitic infection [12]. Treponemal tests such as the *T. pallidum* particle hemagglutination assay (TPHA) and fluorescent treponemal antibody absorption test have higher sensitivity compared with non-treponemal tests (including the VDRL test) but lack specificity for active syphilis infections. Therefore, in cases wherein the CSF-VDRL test is non-reactive, a reactive CSF treponemal test, CSF pleocytosis, or elevated protein levels are able to support the diagnosis of neurosyphilis [13,14].

In the setting of acute ischemic stroke, diagnosing neurosyphilis is challenging because acute stroke itself can cause CSF pleocytosis and elevate protein levels. A study involving patients with acute stroke reported CSF WBC counts of >5 cells/mm^3^ in 12% of patients [15], and the CSF protein levels were reported to be elevated (>45 mg/dL) in 44% of patients [16]. Moreover, CSF protein levels can be elevated in individuals with common conditions such as diabetes mellitus and spinal stenosis [17,18,19]. Magnetic resonance vessel wall imaging (MR-VWI) is a technique that was developed recently that is able to help with the identification of the etiology of stroke. In patients with MVS, MR-VWI findings typically include concentric vessel wall thickening and gadolinium enhancement. Therefore, MR-VWI may be used to confirm the diagnosis of neurosyphilis [20,21]. 

The dramatic nearly sixfold surge in syphilis incidence in Thailand underscores an urgent clinical challenge. This epidemiological shift highlights a significant diagnostic gap, as neurosyphilis is often overlooked in routine stroke workups unless patients lack traditional vascular risk factors. Therefore, evaluating the prevalence in a high-burden setting and investigating the diagnostic utility of advanced imaging like MR-VWI is essential to bridge the gap between systemic serological findings and intracranial vascular pathologies.

The primary objective of this study was to evaluate the prevalence of meningovascular syphilis among patients with acute ischemic stroke or TIA in a tertiary care setting in Thailand. Furthermore, we aimed to assess the diagnostic utility of MR-VWI in identifying vessel wall pathologies in these patients.

## 2. Materials and Methods

In this cross-sectional study, we evaluated the serum and CSF samples of patients aged ≥18 years admitted to the stroke unit of Prapokklao Hospital, Thailand, between March 2022 and October 2022. Patients with stroke mimics and malignant infarction with herniation, and those with a known history of prior or current treatment for syphilis, were excluded from this study. This cross-sectional study was conducted in accordance with the Strengthening the Reporting of Observational Studies in Epidemiology (STROBE) guidelines. The study protocol received formal approval from the Ethics Committee of Chanthaburi Research Ethics Committee/Region 6 (reference number: CTIREC 017/65). All clinical procedures were performed in strict adherence to the relevant institutional and national guidelines and regulations, and written informed consent was obtained from all participants prior to their inclusion in the study.

VDRL (RPR carbon slide agglutination; Cypress Diagnostics, Hulshout, Belgium) and TPHA (SERODIA-TPPA passive particle agglutination test; Fujirebio Inc., Tokyo, Japan) serum tests were performed on samples of all patients to screen for neurosyphilis. If their serum VDRL or TPHA tests were reactive, the patients underwent lumbar puncture to obtain samples for analyzing CSF cell count, protein levels, and VDRL and TPHA reactivity. Subsequently, MR-VWI was performed on patients who had a reactive CSF-VDRL test or a CSF cell count of >5 cell/mm^3^ or a CSF protein level of >50 mg/dL.

### 2.1. Diagnostic Criteria

Patients who had clinical features consistent with MVS and reactive serum serology in at least one of the tests (VDRL or TPHA) were classified into one of the following three cohorts: (1) verified neurosyphilis, if the patient had a reactive CSF-VDRL test [22]; (2) likely neurosyphilis, if the patient had a non-reactive CSF-VDRL, and the CSF WBC count was >5 cell/mm^3^ or the CSF protein level was >50 mg/dL; and (3) positive serology without neurosyphilis, if the CSF-VDRL test was non-reactive, pleocytosis was not detected, and the CSF protein level was ≤50 mg/dL.

For patients who exhibited elevated red blood cell (RBC) counts, WBC counts were corrected using patient’s RBC:WBC count ratio from complete blood count results.

### 2.2. MR-VWI

MR-VWI was performed using Philips Ingenia Prodiva 1.5 Tesla. The study protocol included sagittal T1-weighted (T1W) spin-echo, axial T2W turbo spin-echo (echo time [TE]: 100; repetition time [TR]: 4835; voxel: 0.55 mm × 0.69 mm × 5 mm; slice thickness: 5 mm), axial T1W+contrast (TE: 14; TR: 450; voxel: 0.85 mm × 1.1 mm × 5 mm; thickness: 5 mm), axial diffusion-weighted imaging, three-dimensional (3D) TOF, 3D contrast-enhanced magnetic resonance angiography, pre- and post-contrast T1W 3D brain view (TE: 29; TR: 500; voxel: 1 mm × 1 mm × 1 mm; thickness: 1 mm), and 3D proton density turbo spin-echo (TE: 35; TR: 1300; voxel: 1 mm × 1 mm × 2 mm; thickness: 2 mm). MR-VWI was interpreted by a neuroradiologist with 5 years of experience.

### 2.3. Statistical Analyses 

All data were analyzed using descriptive statistics. Qualitative data (e.g., sex and risk factors) are reported as frequency and percentage. Quantitative data (e.g., age and CSF parameters) are reported as mean ± standard deviation or median and interquartile range, as appropriate, based on data distribution. All analyses were performed using IBM SPSS Statistics for Windows, version 28.0 (IBM Corp., Armonk, NY, USA).

## 3. Results

### 3.1. Patient Characteristics

A total of 400 patients were admitted to the stroke unit of our hospital during the study period. Among them, 34 patients were diagnosed with stroke mimics; thus, 366 patients with ischemic stroke or TIA were included in the analysis (Table 1). The mean patient age was 64.68 years, and 53.3% of the patients were male individuals. The most frequently associated symptoms were dizziness (12.6%) and headache (10.7%). The most common vascular risk factor was hypertension (73.8%), followed by hyperlipidemia (37.7%), diabetes mellitus (30.9%), smoking (20.8%), and atrial fibrillation (9.8%). Interestingly, 20.5% of the patients had a history of stroke or TIA.

### 3.2. Laboratory Analysis

Among the 366 patients included in this study, 21 showed reactivity to either the VDRL or TPHA test (Table 2). Among the 21 patients (5.7%) with a positive screening for syphilis, none showed any reactivity to the CSF-VDRL or TPHA tests, and 8 (2.2%) had a CSF WBC count of >5 cell/mm^3^ or CSF protein level of >50 mg/dL. For two patients, MR-VWI revealed concentric arterial wall thickening and enhancement in the affected middle cerebral arteries.

### 3.3. Demographics and Clinical Characteristics of Patients with Neurosyphilis

Regarding the eight patients who presented CSF pleocytosis or elevated CSF proteins (Table 3), all were male individuals, with an age range of 59–80 (mean 65.6) years, and the majority of them (87.5%) had classic vascular risk factors. None of the patients showed any reactivity to the human immunodeficiency virus test. The majority of patients had no associated symptoms; however, two patients (25%) reported having headaches. MR-VWI was performed in six patients. Two patients (Cases 1 and 2) had middle cerebral artery infarction, with MR-VWI revealing concentric wall thickening enhancement in the bilateral middle cerebral arteries; both patients were diagnosed with likely neurosyphilis. The other four patients had lacunar infarction, with MR-VWI showing eccentric wall thickening enhancement in two patients (Cases 3 and 8) and eccentric wall thickening without enhancement in the remaining two patients (Cases 5 and 6).

## 4. Discussion

In this study, the prevalence of neurosyphilis in patients with acute stroke or TIA who had CSF abnormalities, such as pleocytosis and elevated protein levels, was 2.2%. However, of the six patients in this group who underwent MR-VWI, only two demonstrated concentric wall thickening and enhancement suggestive of neurosyphilis. This finding raises a critical question: do patients who have CSF abnormalities but do not have concentric wall thickening and enhancement on MR-VWI actually have neurosyphilis, or are the CSF abnormalities in such patients merely a non-specific inflammatory response resulting from the acute stroke itself? Furthermore, the visualization of small vessel walls is challenging owing to the limited resolution of MR-VWI [20,21]. The identification of neurosyphilis in our study, despite the presence of traditional vascular risk factors, is particularly significant given the current epidemiological climate in Thailand. The recent, nearly sixfold, surge in syphilis incidence nationwide suggests that our study area is a high-prevalence setting where clinical suspicion for neurosyphilis must be heightened [6]. In such contexts, MVS should no longer be viewed as a rare historical curiosity; rather, it should be perceived as a contemporary diagnostic necessity in the evaluation of acute ischemic stroke. Our findings underscore that failing to perform routine serological screening in these areas inevitably leads to missed diagnoses of a treatable and reversible cause of stroke. It has been reported that the prevalence of neurosyphilis in patients presenting with acute stroke or TIA ranges from 0.3% to 4.7% [23,24,25,26]; however, the actual prevalence of neurosyphilis in these patients remains unknown. There are several reasons for this: Firstly, syphilis serology is not performed on all patients with acute stroke. Secondly, sensitive and specific tests for the diagnosis of neurosyphilis are lacking. Thirdly, the diagnostic criteria for neurosyphilis differ between published studies. Lastly, acute stroke itself can cause CSF pleocytosis and elevate protein levels; hence, if CSF VRDL is non-reactive, diagnosis of neurosyphilis is challenging. 

Traditionally, neurosyphilis is suspected in young patients who have had a stroke; however, our likely neurosyphilis subgroup had a mean age of 65.6 years, consistent with the findings of previous studies [24,26]. This challenges conventional diagnostic assumptions and underscores the fact that neurosyphilis should not be dismissed from differential diagnosis, even in older patients who present with established vascular risk factors. Therefore, not performing serology screening in all patients who have had a stroke may result in missing neurosyphilis diagnosis.

While CSF-VDRL is the definitive diagnostic tool, its suboptimal sensitivity often leads to the production of false-negative results, creating a major diagnostic gap in meningovascular syphilis cases. In high-prevalence areas such as Thailand, MR-VWI emerges as a vital imaging tool to complement inconclusive laboratory data. By visualizing active vessel wall inflammation, MR-VWI provides the objective evidence needed to support a “likely neurosyphilis” diagnosis. Consequently, this allows for the prompt initiation of intravenous penicillin G therapy, potentially improving clinical outcomes in patients who have had a stroke, in whom traditional serological tests fail to provide a clear diagnosis.

There are some limitations to our study that should be acknowledged. Firstly, the number of patients with neurosyphilis was low; secondly, we could not perform MR-VWI in all patients who required MR-VWI according to our study protocol; and thirdly, our study did not utilize polymerase chain reaction (PCR) for the detection of *Treponema pallidum* DNA in the CSF. Although CSF-PCR is a highly specific diagnostic tool, its limited availability in our clinical setting and its variable sensitivity in late-stage neurosyphilis were the primary reasons for its omission. Future studies incorporating PCR alongside advanced neuroimaging might consider offering a more comprehensive diagnostic yield. Finally, we may have missed diagnosing patients with true neurosyphilis in whom only small vessels were affected; this is because, along with MR-VWI with limited resolution, we used a 1.5 Tesla MRI, which produces low-quality images compared with a 3 Tesla MRI. Future large-scale studies are needed to evaluate the MVS diagnostic accuracy of MR-VWI.

In conclusion, in our study, we identified a 2.2% prevalence of likely neurosyphilis among patients with acute ischemic stroke in a regional hospital in Thailand. Given the treatable nature of this condition, systematic serum screening for syphilis is justified in all stroke patients, especially in regions where there is a high prevalence. While traditional CSF-VDRL remains the standard, its potential to yield false-negative results necessitates the use of a comprehensive diagnostic approach. Our findings suggest that MR-VWI serves as a valuable adjunct tool, providing objective evidence of vessel wall inflammation and aiding in the making of clinical decisions when laboratory results are inconclusive. Future larger-scale studies are warranted to further define the role of MR-VWI in the long-term management of meningovascular syphilis.

## Figures and Tables

**Table 1:** Demographics and clinical characteristics of patients with acute ischemic stroke.

Variable	All Patients (*n* = 366)	
Age (years)	64.68 ± 13.6	
<60	130	(35.5)
≥60	236	(64.5)
Sex		
Female	171	(46.7)
Male	195	(53.3)
Associated symptoms		
Headache	39	(10.7)
Dizziness	46	(12.6)
Alteration of consciousness	11	(3.0)
Mood disorder	0	(0.0)
Behavioral change	1	(0.3)
History of fever	4	(1.1)
NIHSS		
At admission	3	(2–5)
At discharge	2	(0–4)
Stroke mechanism		
Small vessel atherosclerosis	159	(43.4)
Large vessel atherosclerosis	144	(39.3)
Cardioembolism	45	(12.3)
Other determined etiology	7	(1.9)
Undetermined etiology	14	(3.8)
Risk factors		
Hypertension	270	(73.8)
Diabetes mellitus	113	(30.9)
Dyslipidemia	138	(37.7)
Atrial fibrillation	36	(9.8)
Smoking	76	(20.8)
Previous stroke/TIA		
No	291	(79.5)
Ischemic	75	(20.5)
HIV		
Non-HIV	11	(3.0)
HIV	3	(0.8)
No test	352	(96.2)

NIHSS, National Institute of Health Stroke Scale; TIA, transient ischemic attack; HIV, human immunodeficiency virus. Data are presented as number (%), mean ± standard deviation, or median (interquartile range).

**Table 2:** Laboratory characteristics of patients seropositive for syphilis.

	Total (*n* = 21)	Likely Neurosyphilis (*n* = 8)	Positive Serology Without Neurosyphilis (*n* = 13)
Positive serum VDRL, *n* (%)	19 (90.5)	7 (87.5)	12 (92.3)
Positive serum TPHA, *n* (%)	20 (95.2)	8 (100.0)	12 (92.3)
Positive CSF-VDRL, *n* (%)	0 (0.0)	0 (0.0)	0 (0.0)
Positive CSF TPHA, *n* (%)	0 (0.0)	0 (0.0)	0 (0.0)
CSF WBC > 5 cell/mm^3^, *n* (%), range (cell/mm^3^)	2 (9.5), 9–10	2 (25.0), 9–10	0 (0.0), 0–4
CSF protein > 50 mg/dL, *n* (%), range (mg/dL)	7 (33.3), 52–108	7 (87.5), 52–108	0 (0.0), 30–49

VDRL, venereal disease research laboratory; TPHA, *Treponema pallidum* hemagglutination assay; WBC, white blood cell.

**Table 3:** Characteristics and high-resolution vessel wall imaging of patients with CSF pleocytosis or elevated CSF protein levels.

Case	Age (Years)	Sex	Risk Factor	Associated Symptoms	NIHSS	Serum		CSF		Infarction Site	MR-VWI
						VDRL	TPHA	WBC (cell/mm^3^)	Protein (mg/dL)		
1	62	M	None	None	4	R	R	2	59.3	Left MCA territory	Concentric wall thickening enhancement at bilateral M1 and M2.
2	59	M	HT	Headache; dizziness	4	R	R	3	53.5	Right MCA territory	Concentric wall thickening enhancement at bilateral M1.
3	70	M	HT	None	1	R	R	0	57.2	Lacunar infarction at left lentiform nucleus and left corona radiata	Focal eccentric wall thickening enhancement at left distal M1.
4	80	M	HT; DM	None	2	R	R	2	80	Left pons	Not performed.
5	61	M	Smoking	None	0	R	R	10	52	Right lentiform nucleus	Eccentric wall thickening at supraclinoid of right ICA without enhancement; multifocal luminal stenosis at bilateral M1.
6	66	M	HT; DM; Smoking	None	0	R	R	0	103	Lacunar infarction at the left lentiform nucleus	Eccentric wall thickening at supraclinoid of right ICA; no wall enhancement.
7	68	M	HT; DM; DLP	Headache	4	NR	R	3	108	Left lentiform nucleus	Not performed.
8	59	M	HT; DLP; Smoking	None	5	R	R	9	41	Lacunar infarction at right caudate nucleus	Multifocal eccentric wall thickening enhancement at right proximal M2 with mild stenosis.

NIHSS, National Institute of Health Stroke Scale; VDRL, venereal disease research laboratory; TPHA, *Treponema pallidum* hemagglutination assay; CSF, cerebrospinal fluid; WBC, white blood cell; MR-VWI, magnetic resonance vessel wall imaging; M, men; NR, non-reactive; R, reactive; MCA, middle cerebral artery; M1, M1 segment of the MCA; M2, M2 segment of the MCA; HT, hypertension; DM, diabetes mellitus; DLP, dyslipidemia; ICA, internal carotid artery.

## Data Availability

The raw data supporting the conclusions of this article will be made available by the authors on request.

## Abbreviations

The following abbreviations are used in this manuscript:

3D	Three-dimensional
CSF	Cerebrospinal fluid
MR-VWI	Magnetic resonance vessel wall imaging
MVS	Meningovascular syphilis
RBC	Red blood cell
STROBE	Strengthening the Reporting of Observational Studies in Epidemiology
T1W	T1-weighted
TE	Echo time
TIA	Transient ischemic attack
TPHA	*Treponema pallidum* particle hemagglutination assay
TR	Repetition time
VDRL	Venereal disease research laboratory
WBC	White blood cell
